# Impact of urinary incontinence on the quality of life (QoL) of women living with HTLV-1 in Salvador, Brazil

**DOI:** 10.1186/1742-4690-11-S1-O17

**Published:** 2014-01-07

**Authors:** Carolina CC de Campos, Ana Karina Galvão-Barroso, Hugo Novais, Marcelo Carvalho, Breno L Araújo, Ney Boa-Sorte, Maria Fernanda Grassi, Ubirajara Barroso, Bernardo Galvão-Castro

**Affiliations:** 1 Bahiana School of Medicine and Public Health, Salvador, Bahia, Brazil; 2 Bahia Federal University, Salvador, Bahia, Brazil; 3 Advanced Laboratory of Public Health, Oswaldo Cruz Foundation, Salvador, Bahia, Brazil

## 

Micturitional alterations are described in women living with HTLV-1. We have determined the prevalence of urinary incontinence types (UI) and evaluated the impact on the quality of life of these women. A cross-sectional study was carried out in Salvador, Brazil from 2009 to 2011. After reviewing the medical records 190 HTLV-1+ women with urinary complains and, aged ≥ 18 were selected. Women with serology positive to HIV and HCV, diabetes mellitus, stroke, Parkinson's disease and cystocele were excluded. 59 women 46 ( 81%) with HAM/TSP and 11(19%) without HAM/TSP were enrolled, 40 (67.8%) answered the Kings Health Questionnaire (KHQ) and 28 (70%) underwent urodynamic evaluation. The average age was 51.2 years. The prevalence of UI was 61.4% and of stress urinary incontinence, urge incontinence and mix urinary incontinence were respectively 16.9%, 35.6% and 47.5%. Detrusor overactivity was detected in 71.4% of women, with 28.6% having sphincter dyssynergia and 7.1% having detrusor hypoactivity. The urge incontinence negatively affected the following items of KHQ: 1) incontinence impact 2) general health perception 3) physical limitation 4) severity measure 5) sleep and disposition 6) emotions 7) limitation on daily activity 8) Social relationships 9) personal relationships. Higher scores in all domains of the mix urinary incontinence were obtained in comparison to urge incontinence. Detrusor overactivity was the alteration that most negatively impacted in all KHQ- QoL domains. Based on the results, it is recommended that all women living with HTLV-1 should be evaluated for micturitional alterations and submitted to urodynamics evaluation when indicated.

